# Active systemic lupus erythematosus is associated with a reduced cytokine production by B cells in response to TLR9 stimulation

**DOI:** 10.1186/s13075-014-0477-1

**Published:** 2014-11-11

**Authors:** Julia Sieber, Capucine Daridon, Sarah J Fleischer, Vanessa Fleischer, Falk Hiepe, Tobias Alexander, Guido Heine, Gerd R Burmester, Simon Fillatreau, Thomas Dörner

**Affiliations:** Department of Medicine/Rheumatology and Clinical Immunology, Charité University Medicine Berlin, CC12, Charitéplatz 01, 10098 Berlin, Germany; German Rheumatism Research Center Berlin (DRFZ), a Leibniz Institute, Charitéplatz 01, 10098 Berlin, Germany; Department of Dermatology, Venerology and Allergology, Allergy-Center-Charité, Charité University Medicine Berlin, Luisenstraße 2, 10117 Berlin, Germany

## Abstract

**Introduction:**

Systemic lupus erythematosus (SLE) is an autoimmune disease associated with a break in self-tolerance reflected by a production of antinuclear autoantibodies. Since autoantibody production can be activated via nucleic acid Toll-like receptor 9 (TLR9), the respective pathway has been implicated in the development of SLE and pathogenic B cell responses. However, the response of B cells from SLE patients to TLR9 stimulation remains incompletely characterized.

**Methods:**

In the current study, the response of B cells from SLE patients and healthy donors upon TLR9 stimulation was analyzed in terms of proliferation and cytokine production and correlated with the lupus disease activity and anti-dsDNA titers.

**Results:**

B cells from SLE patients showed a reduced response to TLR9 agonist compared to B cells from healthy donors in terms of proliferation and activation. B cells from SLE patients with higher disease activity produced less interleukin (IL)-6, IL-10, vascular endothelial growth factor, and IL-1ra than B cells from healthy donors. Further analyses revealed an inverse correlation of cytokines produced by TLR9-stimulated B cells with lupus disease activity and anti-dsDNA titer, respectively.

**Conclusion:**

The capacity of B cells from lupus patients to produce cytokines upon TLR9 engagement becomes less efficient with increasing disease activity, suggesting that they either enter an exhausted state or become tolerant to TLR stimulation for cytokine production when disease worsens.

**Electronic supplementary material:**

The online version of this article (doi:10.1186/s13075-014-0477-1) contains supplementary material, which is available to authorized users.

## Introduction

Systemic lupus erythematosus (SLE) is a severe systemic autoimmune disease with heterogeneous clinical manifestations [1]. A hallmark of SLE immunopathology is B-cell hyperactivity leading to increased numbers of circulating plasma cells [2] and a breakdown of self-tolerance toward DNA and nucleoproteins, which is reflected by elevated levels of antinuclear autoantibodies, such as anti-double-stranded (ds)DNA, anti-ribonucleoprotein and other autoantibodies [3]. In addition, SLE is associated with abnormal cytokine levels, including increased levels of type I interferon (IFN), IL-6, TNF-α, and B-cell activating factor (BAFF), which are thought to have fundamental roles in the maintenance and progression of this inflammatory disease [4-12].

The role of B cells in immunity has been mainly related to the generation of antibodies and formation of immune complexes for a long period of time. However, B cells can exert additional functions, such as antigen presentation, activation of T cells, formation of lymphoid organs and secretion of cytokines, but their contribution in human autoimmunity has not been comprehensively explored [13-16]. However, there is now clear evidence that cytokine-producing B cells can have important roles during autoimmune diseases, suggesting that the role of B cells in SLE pathogenesis might be extended beyond autoantibody production.

It has been shown that cytokine production of B cells can be efficiently induced by toll-like receptor (TLR) signaling [17-19]. In this context, TLR9 is of great interest for SLE immunopathology because increased apoptosis and/or clearance deficiencies in SLE are considered to result in increased amounts of circulating plasma DNA, which may act as TLR agonists and subsequently provide B cell activation signals [20].

Earlier studies showed that SLE B cells responded in a similar way as healthy donors upon TLR9 stimulation. However, B cells from patients with severe SLE showed a reduced secretion of IL-6 and IL-10, and no up-regulation of activation markers, such as CD86 after TLR9 engagement compared to healthy donors [21,22]. To reconcile these findings, we undertook a more comprehensive study of cytokine production by B cells in SLE. The current study compared B cells from healthy donors and SLE patients for production of cytokines and growth factors, proliferation and expression of activation markers upon TLR9 stimulation taking the underlying lupus activity into consideration.

## Materials and methods

### Patients and controls

For the analysis of cytokine production by B cells, peripheral blood was collected from 18 SLE patients (17 females/1 male) with a mean age of 34.9 ± 10.4 years and 13 healthy donors (12 females/1 male) with a mean age of 36.7 ± 14.9 years. For the analysis of activation and IL-10 expression in B cells using flow cytometry (FC), peripheral blood was collected from 6 female SLE patients with a mean age of 38.8 ± 12.9 years and 10 healthy donors (8 female/2 male) with a mean age of 32.9 ± 11.1 years. For the analysis of TLR9 expression, peripheral blood was collected from patients with SLE (12 female/1 male, 38.4 ± 18.4) and 5 female healthy donors (29.4 ± 5.0).

The study was approved by the local ethics committee of the *Charité Universitätsmedizin* Berlin and written consent was obtained from all donors. The consents are on file held by the principal investigator and available for review by the editor-in-chief upon request. All patients met the revised American College of Rheumatology classification criteria for SLE [23]. The disease activity was assessed using the SLE disease activity index (SLEDAI) modified according to the SELENA-trial [24]. Details of the clinical characteristics and treatment regimens of the analyzed SLE patients are provided in Table 1.

**Table 1:** **Demographic and clinical data, lupus activity (SLEDAI) and individual therapy of the patients at the time of analysis**

**Patient ID**	**Sex**	**Age, y**	**B cells/μL blood**	**anti-dsDNA IgG, U/mL**	**SLEDAI**	**Treatment**	**Prednisolone dose, mg/d**
SLE1	^F^	61	187	0	7	Pred	5.0
SLE2	^F^	31	320	0	5	Pred, MTX	5.0
SLE3	^F^	30	159	0	4	Pred, MMF	5.0
SLE4	^F^	25	132	45	8	Pred, Aza, HCQ	5.0
SLE5	^F^	36	216	38	8	Pred, Aza	7.5
SLE6	^F^	39	538	50	6	Pred, MMF	5.0
SLE7	^F^	22	164	0	4	Pred	5.0
SLE8	^F^	44	108	0	5	HCQ	0
SLE9	^F^	48	67	75	8	Pred, MMF	5.0
SLE10	^M^	20	89	50	10	Pred, Aza	5.0
SLE11	^F^	30	305	45	6	Pred	5.0
SLE12	^F^	33	468	0	5	Pred	8.0
SLE13	^F^	23	785	125	14	Pred, HCQ	100.0
SLE14	^F^	33	110	18	4	Pred, HCQ	5.0
SLE15	^F^	32	75	60	12	Pred, MMF	8.0
SLE16	^F^	37	ND	2,000	15	Pred, HCQ	25.0
SLE17	^F^	38	192	21	8	Pred, HCQ, MMF	7.5
SLE18	^F^	47	114	1,400	14	Therapy naive	0
SLE19	^F^	59	26	0	5	Pred, Aza	5.0
SLE20	^F^	30	32	1,000	5	HCQ	0
SLE21	^F^	51	81	200	6	Pred, Aza	7.5
SLE22	^F^	28	56	0	6	Ciclosporine, Pred	5.0
SLE23	^F^	33	98	0	6	Pred, Aza	5.0
SLE24	F	32	155	17.5	18	Cylophosphamide, Pred	7.5
SLE25	F	29	19	positive	7	Ciclosporine, Pred	-
SLE26	^M^	21	79	positive	6	Pred, MMF	-
SLE27	^F^	37	118	positive	5	Pred, Aza	-
SLE28	^F^	74	20	negative	5	Antimalarials	-
SLE29	^F^	35	75	positive	6	Pred, Antimalarials	-
SLE30	^F^	33	35	positive	7	Pred, MMF	-

Patient SLE18 was newly diagnosed and donated blood before immunosuppressive treatment was started. SLEDAI, systemic lupus erythematosus disease activity index; Pred, prednisolone; Aza, azathioprine; MTX, methotrexate; HCQ, hydroxychloroquine; MMF, mycophenolate mofetil; ND, not detected.

### Isolation of B cells

Peripheral blood mononuclear cells (PBMCs) were isolated with density gradient centrifugation using lymphocyte separation medium (PAA Laboratories, Pasching, Austria) as previously described [25]. Subsequently, B cells were negatively purified by magnetic activated cell sorting (MACS®) using the B-cell Isolation Kit II (Miltenyi Biotec, Bergisch Gladbach, Germany) according to the manufacturer’s instructions and B cell purity was checked by flow cytometry. The contamination with CD3^+^, CD14^+^ and dead cells was below 5% in all samples.

### Lymphocyte staining for flow cytometry

Purified B cells were stained at 4°C for 15 minutes with antibodies against CD14-PB (M5E2), CD3-PB (UCHT1), CD27-Cy5 (2E4), CD19-PE-Cy7 (SJ25C1), CD20-PerCP-Cy5 (L27), and IgD-FITC (IA62) to control the purity used for the subsequent analyses.

Before and after stimulation, PBMCs were stained first with antibodies against CD14- Pacific blue (PB) (M5E2), CD3-PB (UCHT1), CD27-fluorescein isothiocyanate (FITC) (L128), CD38-PercP-Cy5.5 (HIT2) and CD20-Pacific orange (PO) (H147) for 10 minutes on ice. After washing, PBMCs were incubated with 400 μl of 1 × FACS permeabilizing solution 2 (Becton Dickinson (BD) Franklin Lakes, NJ, USA) for 10 minutes at room temperature (RT). After permeabilization and washing, PBMCs were stained with anti-Ki67-PE-Cy7 (B56) and anti-IL-10-APC (JES3-9D7) and anti-TLR9-PE (eB72-1665) antibodies for 10 minutes at RT. All antibodies were purchased from BD; beside Cy5-conjugated anti-CD27 antibody (2E4) (kind gift from Andreas Thiel, Berlin Center for Regenerative Therapy, Charité Berlin) and anti-IL-10-APC antibodies purchased from Miltenyi Biotec. Stained cells were analyzed by FC using the FACSCanto™II flow-cytometer (BD). FC data were analyzed using FlowJo (Tree Star, Inc., Ashland, OR, USA).

### *In vitro* stimulation

B cells were stimulated *in vitro* with CpG 2006 oligonucleotide (CpG) (TIB MolBiol Synthese Labor GmbH, Berlin, Germany). The cells were resuspended in RPMI 1640 Glutamax supplemented with 10% FCS (Lonza, Köln, Germany), 5% penicillin/streptomycin, and 0.05 mM 2-mercaptoethanol (Gibco® Life Technologies GmbH, Darmstadt, Germany). B cells, 10^5^, were seeded and stimulated with 2.5 μg/mL CpG for 48 h at 37°C and 5% CO_2_. After 2 days of culture, the supernatants were harvested and frozen at −70°C prior to analysis.

To analyze the IL-10 production by B cells, PBMCs (10^6^/well) were cultured with CpG 2006 *in vitro* as described [26,27]. Intracellular staining of IL-10 and Ki67 was performed on PBMCs after 2 days of culture. PBMCs were re-stimulated for 4 h with 10 ng/mL PMA and 1 μM ionomycin including 2 μg/mL brefeldin A for the last 2 h (all from Sigma Munich, Germany) prior to intracellular staining. Unstimulated cells served as controls.

### Cytokine assay

Cryopreserved supernatants were assessed for determination of cytokine concentration using Bio-Plex® technology (Bio-Rad Laboratories, Inc., CA, USA) according to the manufacturer’s instructions. The cytokines analyzed were IL-1β, IL-1ra, IL-2, IL-4, IL-5, IL-6, IL-7, IL-8/CXCL8 (chemokine (C-X-C motif) ligand 8), IL-9, IL-10, IL-12p70, IL-13, IL-15, IL-17A, eotaxin-1/CCL11 (chemokine (C-C motif) ligand 11), basic fibroblast growth factors (FGF), granulocyte colony-stimulating factor (G-CSF), granulocyte macrophage colony-stimulating factor (GM-CSF), IFN-α2, IFN-γ, IP-10 (IFNγ-induced protein 10)/CXCL10, monocyte chemotactic protein-1 (MCP-1)/CCL2, macrophage inflammatory protein-1α (MIP-1α)/CCL3, MIP-1β/CCL4, platelet-derived growth factor-BB (PDGF-BB), regulated on activation, normal T-cell expressed and secreted (RANTES)/CCL5, vascular endothelial growth factor (VEGF) and TNF-α. The assay sensitivity depends on the particular cytokines analyzed from 0.3 pg/mL for IL-10 to 6.4 pg/mL for IFN-γ. Although all 28 cytokines and growth factors were detectable in the supernatant of the B-cell culture; 12 of them (IL-1β, IL-2, IL-4, IL-5, IL-7, IL-12p70, IL-13, IL-15, GM-CSF, IFN-α2, eotaxin-1, and MCP-1) were produced at low levels with a mean concentration below 20 pg/mL after TLR9 stimulation (Additional file 1) and were therefore not considered for further analysis.

### Statistical analysis

The statistical analysis was performed with SPSS (version 20, IBM, NY, Chicago, IL, USA). To compare data from healthy donors and SLE patients, the nonparametric Mann-Whitney *U*-test was used. The Wilcoxon test was used to compare results after TLR9 stimulation with unstimulated controls for the cytokine production. Multiple comparisons were performed using one way analysis of variance (ANOVA) with Dunnett’s post hoc test. To correlate cytokine levels with SLEDAI scores or with dsDNA titers, Spearman correlation analysis was performed. *P*-values <0.05 were considered statistically significant. The statistical tests used are indicated in each figure legend.

## Results

In the current study, a comprehensive analysis was performed to assess the capacity of B cells from SLE patients to respond to TLR9 stimulation in terms of proliferation, activation, and cytokine production in relation with clinical lupus activity using SLEDAI.

### B cells from SLE patients have a reduced proliferation and activation upon TLR9 stimulation

**Figure 1:**
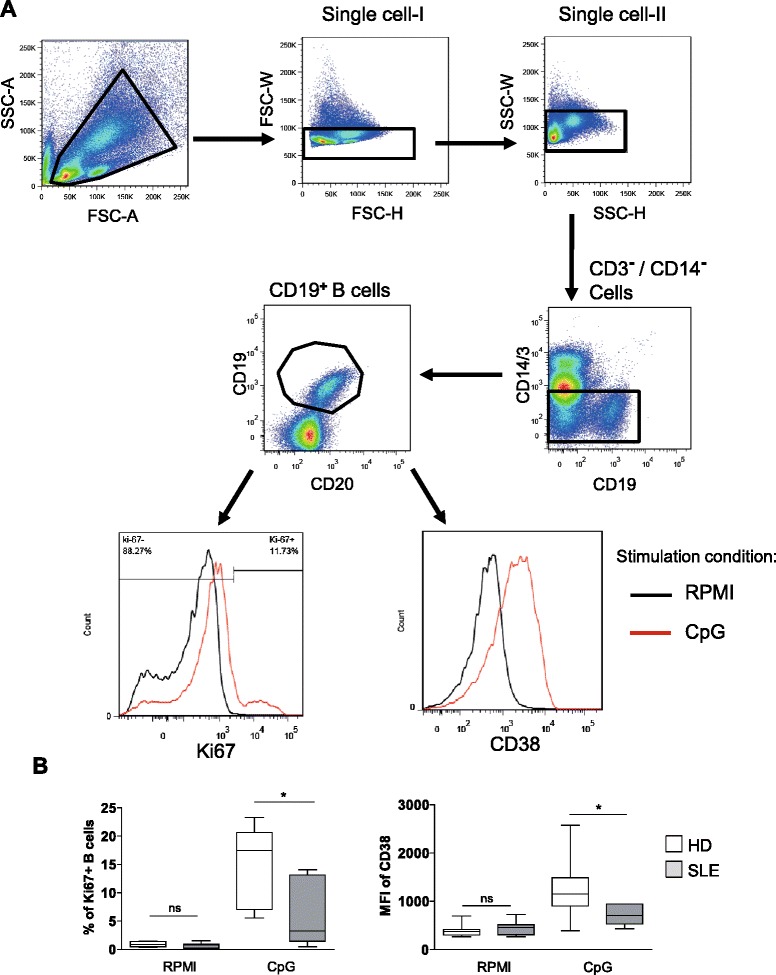
**Reduced proliferation and activation of toll-like receptor 9 (TLR9)-stimulated B cells from systemic lupus erythematosus (SLE) patients compared to healthy donors. (A)** Representative flow cytometry plots showing the gating strategy and histograms of the frequency of proliferating B cells and induction of CD38 expression after 2 days of peripheral blood mononuclear cells (PBMC) culture with or without CpG stimulation. **(B)** Combined data from 6 SLE patients and 10 healthy donors for the frequency of proliferating (Ki-67^+^) B cells (left graph) and the induction of CD38 expression by B cells (right graph). (Mann-Whitney *U*-test; ns, not significant *P <0.05). HD, healthy donors.

We first evaluated the response of B cells to TLR9 stimulation in terms of proliferation and activation. The frequency of proliferating cells (% of Ki67^+^ B cells), and the upregulation of the activation marker CD38 after 2-day culture with CpG were analyzed by FC (Figure 1A). CpG induced B cell proliferation independently of B cell receptor (BCR) engagement; however, SLE patients had a lower frequency of proliferating B cells upon TLR9 stimulation in comparison with healthy donors (*P* <0.05) (Figure 1B, left graph). Moreover, activation of B cells was evaluated by upregulation of CD38 expression (mean fluorescence intensity, MFI) upon TLR9 stimulation. B cells from healthy donors significantly upregulated CD38, resulting in a 3-fold increase after TLR9 stimulation, while the response of B cells from SLE patients was significantly lower (Figure 1B, right graph, *P* <0.05).

### B cells from SLE patients secrete fewer cytokines in relation to the disease activity

**Figure 2:**
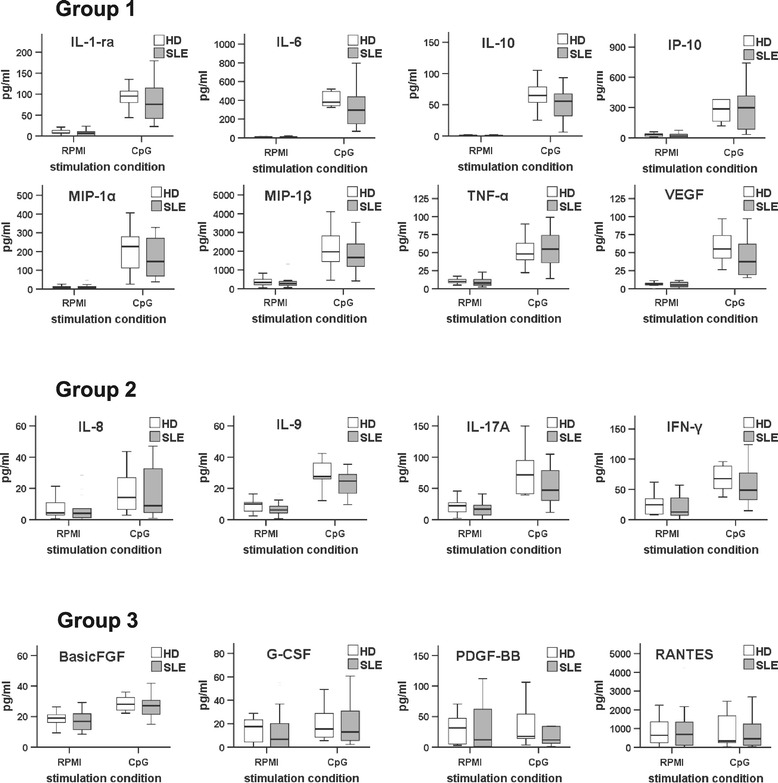
**Response to toll-like receptor 9 (TLR9) stimulation of B cells from systemic lupus erythematosus (SLE) patients and healthy donors.** B cells from SLE patients and healthy donors were purified and cultured with or without CpG. After 2 days of culture, the supernatants were collected and cytokine concentrations were measured by bioplex. Group 1 consists of certain cytokines with strong increase (>4-fold) upon TLR9 stimulation of B cells. Group 2 is composed of cytokines with a moderate increase (between 2- and 4-fold) upon TLR9 stimulation, while group 3 reflects cytokines with a very limited increase (<2 fold) after TLR9 stimulation (n =18 SLE patients). FGF, fibroblast growth factor; G-CSF, granulocyte colony-stimulating factor; G-CSF, granulocyte colony-stimulating factor; IP-10: IFNγ-induced protein 10; MIP, macrophage inflammatory proteins; PDGF-BB, platelet-derived growth factor-BB; RANTES, regulated on activation: normal T-cell expressed and secreted; VEGF, vascular endothelial growth factor.

**Figure 3:**
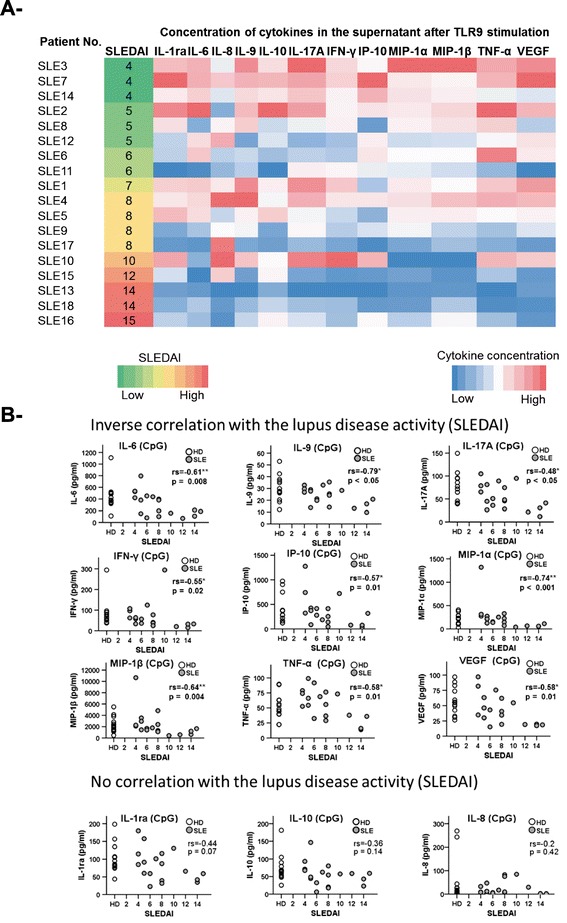
**Hyporesponsiveness to toll-like receptor 9 (TLR9) stimulation of B cells from systemic lupus erythematosus (SLE) patients in relation to their disease activity. (A)** Heat map of cytokines from group 1 and 2 secreted by B cells upon TLR9 stimulation (dark blue for the lowest, to red for the highest concentration of cytokine in the supernatant). SLE patients were ordered according to their disease activity from low systemic lupus erythematosus disease activity index (SLEDAI) in green, to high SLEDAI in red. **(B)** Direct correlation between individual cytokines from group 1 and 2 and lupus activity (SLEDAI score). Significant inverse correlation was found between the SLEDAI score and individual cytokines produced by B cells upon TLR9 stimulation (IL-6, IL-9, IL-17A, IFN-γ, IP-10, MIP-1α, MIP-1β, and TNF-α). Healthy donors (HD) are plotted as reference. (Spearman *r* correlation test; *P <0.05 and **P <0.01). IP-10: IFNγ-induced protein 10; MIP, macrophage inflammatory proteins; VEGF, vascular endothelial growth factor.

Subsequently, the influence of TLR9 stimulation on cytokine production was evaluated by analyzing the concentrations of 28 cytokines and growth factors in the supernatants of B cell cultures from healthy donors and SLE patients using BioPlex technology. The cytokines were grouped according to the level of their induction upon TLR9 engagement compared to unstimulated B cells. The first group included cytokines showing more than a 4-fold increase after TLR9 stimulation and comprised IL-1ra, TNF-α, IL-6, and IL-10, the chemokines IP-10, MIP-1α, -1β, and the growth factor VEGF. The second group was defined by a moderate (2- to 4-fold) increase after TLR9 stimulation, and comprised IL-8, IL-9, IL-17A and IFN-γ. A third group of growth factors and chemokines, defined by a very small increase or no increase after TLR9 stimulation (maximum 2-fold increase) comprised basic FGF, G-CSF, PDGF-BB, and RANTES (Figure 2). Overall, the profiles of cytokine secretion observed in the supernatants of cultured B cells from SLE patients and healthy donors shared very large similarities. We found that none of the cytokines from groups 1 and 2 were secreted at a higher level by B cells from SLE patients, but rather at lower levels in comparison to B cells from healthy donors. Since earlier reports found that B cells from active SLE patients were less responsive to TLR9 stimulation in terms of IL-6 and IL-10 production [21], we analyzed the relation between the cytokines from groups 1 and 2 and the disease activity (SLEDAI) by using a heat map (Figure 3A). When the patients were ordered according to their SLEDAI, it became apparent that B cells from patients with SLEDAI of 4 (n = 3) produced larger amounts of cytokines than those from patients presenting with a SLEDAI higher than 14 (n = 3). The remaining patients with a SLEDAI between 4 and 14 displayed an intermediate but clearly ranked profile (Figure 3A). In greater detail, correlation analyses of individual cytokines revealed significant inverse correlations between the SLEDAI and inducible amounts of IL-6, IL-9, IL-17A, IFN-γ, IP-10, MIP-1α, MIP-1β, TNF-α, and VEGF of TLR9-activated SLE B cells (Figure 3B). In contrast, there was no correlation between the spontaneous (unstimulated) production of these cytokines and the SLEDAI, highlighting the specificity of this association with TLR9 signaling.

**Figure 4:**
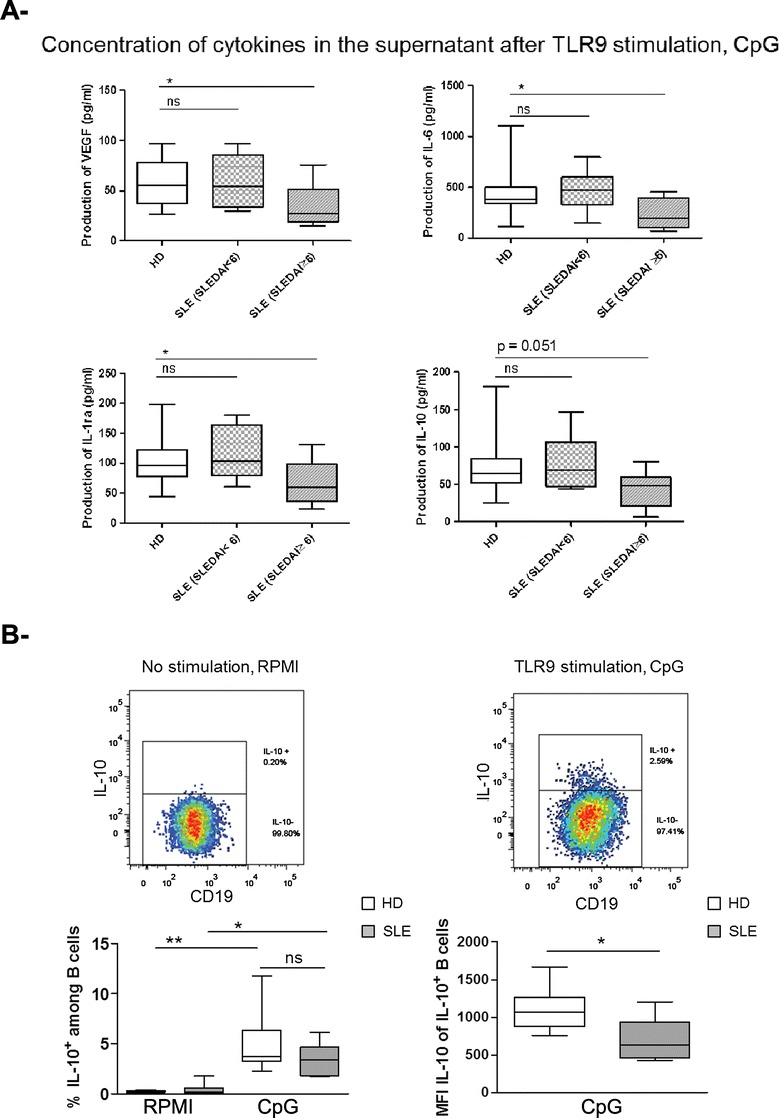
**Reduced IL-6, vascular endothelial growth factor (VEGF), and IL-1ra production by B cells upon toll-like receptor 9 (TLR9) stimulation in active systemic lupus erythematosus (SLE) patients compared to healthy donors (HD). (A)** To compare the production of cytokines (group 1 and 2) upon TLR9 stimulation by healthy donors with SLE patients, the SLE cohort was divided in two groups with low disease activity (SLEDAI <6, n = 6) and high disease activity (SLEDAI ≥6, n = 12). IL-6, VEGF, and IL-1ra produced by B cells upon TLR9 stimulation were significantly reduced in SLE with high SLEDAI compared to healthy donors (one way analysis of variance with Dunnett’s post hoc test, *P <0.05). **(B)** Representative flow cytometry analysis plots showing IL-10-producing B cells after 2 days of peripheral blood mononuclear cells (PBMC) culture without (left) or with (right) CpG stimulation. Combined data from 6 SLE patients and 10 healthy controls for the frequency of IL-10-producing B cells (left graph) and the overall production of IL-10 by B cells (right graph, MFI = mean fluorescence intensity, reflecting amount per cell) (Mann-Whitney *U*-test and Wilcoxon test; ns: not significant, *P <0.05, **P <0.01).

This global analysis showed that TLR9-stimulated B cells from patients with active SLE produced fewer cytokines than those from patients with less active disease. In order to further compare the data to healthy donors, we divided the patients into two groups with high (≥6) and low SLEDAI (<6) [28] (Figure 4A). Notably, the production of IL-6, IL-1ra, and VEGF by B cells was significantly reduced in patients with active SLE with a SLEDAI ≥6 compared to healthy donors. The secretion of IL-10 was also reduced by trend, although not statistically significant (*P* = 0.051). FC analysis of IL-10-producing B cells showed a significantly increased frequency of IL-10^+^ B cells after TLR9 stimulation (Figure 4B). However, there was no difference between B cells from SLE patients and healthy donors. While the generation of IL-10-producing B cells was not reduced in culture of B cells from SLE patients, there was a clear reduction of the amount of IL-10 induced in individual IL-10^+^ B cells compared to healthy donors as shown by the MFI of intracellular IL-10 (Figure 4B).

### Inverse correlation between cytokine production and anti-dsDNA autoantibodies

**Figure 5:**
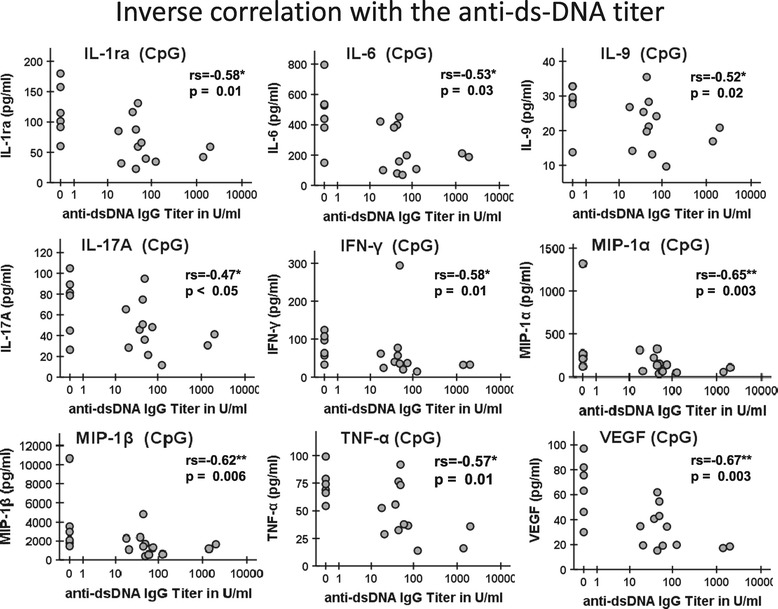
**Reduced cytokine production by B cells after toll-like receptor 9 (TLR9) stimulation in patients with higher anti-dsDNA antibody titers.** The anti-dsDNA antibodies were measured in the serum of the patients simultaneously with the TLR9 activation of B cells. There were a number of significant inverse correlations between serum anti-dsDNA-antibody titers and TLR9 induced cytokines (IL-1ra, IL-6, IL-9, IL-17A, IFN-γ, MIP-1α, MIP-1β, TNF-α, and VEGF) in SLE patients (Spearman *r* correlation test, *P* <0.05 and **P <0.01). MIP, macrophage inflammatory proteins; VEGF, vascular endothelial growth factor.

A key serologic parameter reflecting the breakdown of tolerance and related with lupus disease activity are anti-dsDNA autoantibodies that have been linked to TLR9 stimulation [29]. We found a significant (*P* <0.05) inverse correlation between the serum anti-dsDNA titers and amounts of IL-1ra, IL-6, IL-9, IL-17A, IFN-γ, MIP-1α, -1β, TNF-α, and VEGF produced by TLR9-activated B cells from SLE patients (Figure 5). Thus, the higher anti-dsDNA antibody titer in serum, the lower was the level of these cytokines produced *in vitro* by CpG activated B cells.

### TLR9 is downregulated in patients with active SLE

**Figure 6:**
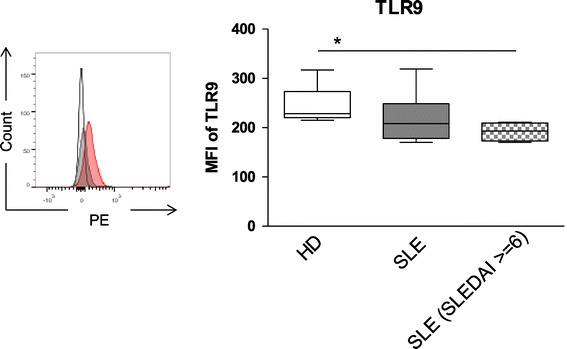
**Reduced expression of toll-like receptor 9 (TLR9) by B cells from active systemic lupus erythematosus (SLE) patients.** Representative flow cytometry histogram showing TLR9 staining for B cells (left). Combined data for TLR9 detection of B cells obtained from 6 SLE patients and 4 healthy donors (HD), respectively, did not show a significant difference in TLR9 detection, but B cells from patients with more active SLE (SLE disease activity index (SLEDAI) ≥6) were found to express reduced TLR9 (Mann-Whitney *U*-test; ns: not significant; *P <0.05) (right panel). Hhistogram: red line TLR9, gray line isotype- phycoerythrin (PE) and black line control antibody; MFI: mean fluorescence intensity of TLR9.

In an attempt to explain the reduced response of B cells from SLE patients to TLR9 agonist compared to controls, we analyzed whether TLR9 was differentially expressed by these cells. FC analysis of TLR9 expression in B cells from healthy donors and SLE patients showed that the MFI of TLR9 was similar in B cells from healthy donors compared to those from SLE patients (242.8 ± 41.9 and 218.5 ± 53.2) (Figure 6). However, a significant reduction of TLR9 expression was found for B cells from SLE patients with a SLEDAI ≥6 (191.8 ± 19.5) (Figure 6). This result is in line with our observations on cytokine production, suggesting that the observed reduction in CpG responsiveness for B cells from patients with high disease activity could be related to a down-modulation of TLR9 expression.

## Discussion

Our results confirm that B cells from SLE patients globally show the same pattern of cytokine expression compared to B cells from healthy donors upon TLR9 stimulation [30]. However, our study also provides evidence that B cells from patients with severe SLE are hyporesponsive to TLR9 stimulation in terms of activation, proliferation, and cytokine production compared with B cells from healthy donors. Moreover, the reduction of cytokine production upon TLR9 stimulation was correlated with lupus activity and anti-ds-DNA antibody titers. Patients with high SLEDAI score (SLEDAI ≥6) showed a lower secretion of IL-6, IL-1ra, IL-10, and VEGF upon TLR9 stimulation in comparison to healthy donors as well as a lower expression of TLR9. Of note, a previous report documented that SLE B cells expressed significantly more TLR9 than B cells from healthy donors [31], especially in patients with increased anti-dsDNA antibody titers and high disease activity [32,33]. Although the reason for the discrepancy between our results and this study remains unexplained, it seems coherent that B cells from patients with active SLE have both reduced TLR9 expression and a lower response to TLR9 agonist than B cells from healthy donors. Remarkably, we did not identify any cytokine that was induced to a larger extent in B cells from SLE patients compared to healthy donors.

In this study the mechanism responsible for lower expression of TLR9 by B cells from patients with active SLE remains to be delineated. A possibility is that B cells from patients with severe SLE are hyporesponsive to TLR9 stimulation because of an overstimulation *in vivo* by circulating DNA in the serum of the patients [21]. This might indicate an exhausted or post-activation state as already described for T cells from SLE patients [1], or a state of tolerance to TLR-stimulation as described for myeloid cells [34]. Another reason might be that TLR9 signaling limits the life span of anti-DNA B cells, leading to an elimination of the B cells expressing high amounts of TLR9, as shown in an SLE mouse model [35]. In any case, such a loss of TLR9 responsiveness might represent an attempt of the immune system to reduce the availability of this potentially deleterious pathway as the disease worsens. Alternatively, intrinsic TLR9 signaling in B cells might be beneficial in SLE so that the observed impairment could play a role in exacerbation of the disease. It is currently difficult to evaluate whether intrinsic TLR9 signaling in B cells is beneficial or deleterious during chronic SLE. TLR9-stimulated B cells secreted inflammatory cytokines, such as IL-6, TNF-α, MIP-1α, -1β, and IP-10, but also anti-inflammatory cytokines such as IL-1ra [36], and IL-10 [13,37]. We found reduced IL-10 production by B cells upon TLR9 stimulation for active SLE patients, consistent with previous reports [21]. IL-1ra and VEGF have also been described to have immunosuppressive effects, and were also produced in lower amounts by B cells from active patients compared to healthy donors. Indeed, IL-1ra is a receptor antagonist that inhibits the IL-1 pathway and provides an important anti-inflammatory mechanism [38]. VEGF has also been described as an immunosuppressive cytokine that inhibits the functional maturation of dendritic cells and T-cell development [39,40]. The link between SLE pathogenesis and the reduced expression of IL-1ra and VEGF by B cells remains to be further analyzed.

The capacity of B cells to produce cytokines can be influenced by a number of variables in addition to the disease activity, including immune therapies. In this regard, the current data do not indicate that certain therapies (Table 1) may have major effects on cytokine-production by B cells upon stimulation *in vitro*. In particular there was no significant difference in the amount of cytokines produced by B cells from patients treated with hydroxychloroquine (n = 6) or not (n = 12) (data not shown), although this drug is considered to inhibit TLR9-signaling [41]. We also correlated the dosage of prednisolone (mg/day) taken by the patient at the moment of the study and the cytokine level produced by B cells upon TLR9 stimulation (data not shown). Only the concentration of IL-9, IL-17A, IFN-γ and RANTES showed a significant inverse correlation with the dosage of prednisolone used by the patients. Nevertheless, only two patients received more than 10 mg prednisolone/day in this study. In addition, one newly diagnosed and untreated patient (SLE18) with a SLEDAI of 14 showed a very low cytokine production, in a range comparable to the other patients with active SLE who were under immunosuppressive treatment. Thus, the described low cytokine production upon TLR9 stimulation by B cells of active patients seems related to SLE disease activity *per se* rather than to immunosuppressive interventions.

## Conclusion

The cytokine production by B cells from patients with severe SLE upon TLR9-engagement *ex vivo* is substantially lower than in healthy donors. The current data are consistent with an exhaustion of B cells, or an induction of TLR-tolerance post-activation (by diminished TLR9 expression) depending on lupus disease activity. Understanding the molecular mechanism of reduced cytokine production by B cells upon TLR9 engagement in SLE might provide new insights into the pathogenesis of SLE.

## Additional file

Additional file 1: Figure S1 Cytokines lacking substantial production upon toll-like receptor 9 (TLR9) stimulation by B cells from healthy donors (white) and patients suffering from systemic lupus erythematosus (SLE) (gray). B cells from SLE-patients and healthy donors were purified and cultured with or without CpG, the cytokine concentrations were measured in supernatants collected after 2 days of culture by bioplex. The cytokines shown were detectable at very low levels after B-cell stimulation by TLR ligation using CpG (<20 pg/mL of supernatant).

## Abbreviations

Aza: azathioprine
BAFF: B-cell activating factor
Basic FGF: fibroblast growth factors
BCR: B cell receptor
BD: Becton Dickinson
CCL: chemokine (C-C motif) ligand
CXCL: chemokine (C-X-C motif) ligand
FC: flow cytometry
FITC: fluorescein isothiocyanate
G-CSF: granulocyte colony-stimulating factor
GM-CSF: granulocyte macrophage colony-stimulating factor
HCQ: hydroxychloroquine
IFNm: interferon
IL: interleukin
IP-10: IFNγ-induced protein 10
MCP-1: monocyte chemotactic protein-1
MIP: macrophage inflammatory proteins
MMF: mycophenolate mofetil
MTX: methotrexate
PB: Pacific blue
PBMCs: peripheral blood mononuclear cells
PDGF-BB: platelet-derived growth factor-BB
PE-Cy: phycoerythrin-cyanin
PerCP: peridinin-chlorophyll protein
PO: Pacific orange
Pred: prednisolone
RANTES: regulated on activation, normal T-cell expressed and secreted
SLE: systemic lupus erythematosus
SLEDAI: systemic lupus erythematosus disease activity index
TLR9: toll-like receptor 9
TNF: tumor necrosis factor
VEGF: vascular endothelial growth factor
