# Supplementary material for: Active systemic lupus erythematosus is associated with a reduced cytokine production by B cells in response to TLR9 stimulation
Source: Arthritis Res Ther. 2014 Nov 11;16(6):477. doi: 10.1186/s13075-014-0477-1 (PMC4247768; doi:10.1186/s13075-014-0477-1)
Supplement: Additional file 1: Figure S1 — Cytokines lacking substantial production upon toll-like receptor 9 (TLR9) stimulation by B cells from healthy donors (white) and patients suffering from systemic lupus erythematosus (SLE) (gray). B cells from SLE-patients and healthy donors were purified and cultured with or without CpG, the cytokine concentrations were measured in supernatants collected after 2 days of culture by bioplex. The cytokines shown were detectable at very low levels after B-cell stimulation by TLR ligation using CpG (<20 pg/mL of supernatant). [file 13075_2014_477_MOESM1_ESM.pdf]

Additional file 1

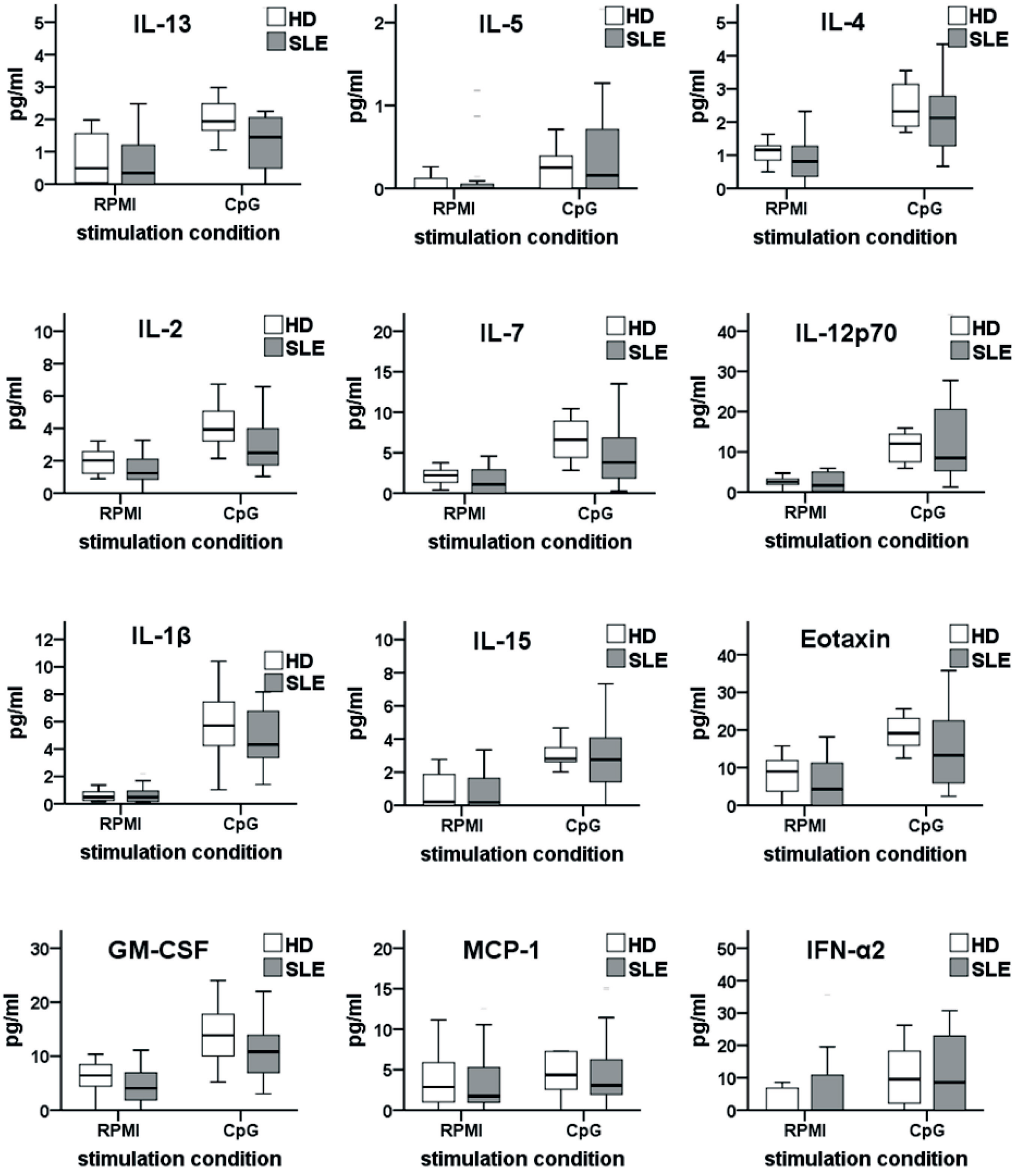

Cytokines lacking substantial production upon TLR9 stimulation by B cells from healthy donors (white) and patients suffering from SLE (grey)

B cells from SLE-patients and healthy donors were purified and cultured with or without CpG, the cytokine concentrations were measured in supernatants collected after 2 days of culture by bioplex. The cytokines shown were detectable at very low levels after B-cell stimulation by TLR ligation using CpG (<20pg/mL of supernatant).
